# A cross-sectional study to evaluate metabolic and demographic factors affecting cognitive function among low educated internal medicine outpatients

**DOI:** 10.17712/nsj.2017.1.20160259

**Published:** 2017-01

**Authors:** Feride Alakus, Serife A. Helvaci, Mustafa Temizel, Yucel Arman

**Affiliations:** *From the Internal Medicine Clinics (Alakus, Arman, Helvaci), Okmeydani Training and Research Hospital, and the Department of Internal Medicine (Temizel), Medicana International Hospital, Istanbul, Turkey*

## Abstract

**Objective:**

To evaluate factors affecting cognitive function in internal medicine outpatients.

**Methods:**

A total of 130 consecutive outpatients aged 50-80 years old were included in this cross-sectional study conducted at Okmeydani Training and Research Hospital, Istanbul, Turkey between March and May 2013. Cognitive function was evaluated via Standardized Mini-Mental State Examination (SMMSE) scores. Logistic regression analysis was performed to determine factors predicting poor cognitive function.

**Results:**

Mild-to-moderate cognitive impairment was noted in 39.2% of the patients. Median (interquartile range) total SMMSE scores were significantly higher in patients aged ≤60 than >60 years (27.0 (2.0) vs. 25.0 (5.0), *p*=0.000). Multivariate linear regression analyses revealed female gender (B, −1.27; 95% CI, −2.36 to −0.18; *p*=0.023) and aging (B, −0.20; 95% CI, −0.26 to −0.14; *p*<0.001) to result in a significant decrease in the total SMMSE scores.

**Conclusions:**

Mild-to-moderate cognitive impairment was observed in 39.2% of internal medicine outpatients. Old age and female gender were significant predictors of lower total SMMSE scores. Furthermore, besides for high language scores in hypertensive patients on combined therapy, no significant impact of hypertension or obesity was observed on the SMMSE scores.

The clinical syndrome of dementia is characterized with cognitive impairment categorized into different domains including memory, executive function, language, visuospatial abilities, and personality and behavior.^1^,^2^ Given the significant clinical and pathological coincidence of categorical domains and the progressive cognitive decline with involvement of more domains in dementia, distinguishing different etiologies of dementias is considered challenging, particularly in the later stages.^1^-^3^ Standardized Mini-Mental State Examination (SMMSE) has been used to detect cognitive disorders and monitor treatment response in dementia syndromes in clinical practice as well as in epidemiological research involving elderly people.^4^,^5^ The SMMSE is used globally to determine cognitive ability along with its limited potential to differentiate among clinical syndromes.^6^,^7^ The present study was designed to evaluate factors affecting cognitive function based on SMMSE among Internal Medicine Outpatients.

## Methods

### Study population

A total of 130 consecutive outpatients aged 50-80 years old [mean±standard deviation (SD), 61.7±8.7 years old; 63.1% females], who were admitted to Internal Medicine Outpatient Clinics at Okmeydani Training and Research Hospital, Istanbul, Turkey between March and May 2013 were included in this cross-sectional study. Exclusion criteria comprised the presence of dementia, parkinson’s disease, multiple sclerosis, and hyperlipidemia; history of cerebrovascular accident or psychiatric disease; treatment with drugs used to ameliorate psychiatric conditions; use of antihypertensive medications as a secondary prevention for cardiovascular disease; and presence of chronic kidney disease, thyroid disease, vitamin B12 or folic acid deficiency, severe anemia (hemoglobin levels <8 g/dL), or diabetes mellitus. Written informed consent was obtained from each subject following a detailed explanation of the objectives and protocol of the study, which was conducted in accordance with the ethical principles stated in the “Declaration of Helsinki” and approved by the Okmeydani Training and Research Hospital Ethics Committee (Date of Approval 09-Feb-2013, protocol no. 60).

### Study parameters

Data on demographics, body mass index (BMI; kg/m^2^), hypertension and cognitive ability based on the SMMSE scores were recorded for each patient via a face-to-face interview. Association between the SMMSE scores and demographic and clinical parameters was evaluated, and logistic regression analysis was performed to determine factors predicting poor cognitive function.

### Blood pressure (BP) measurement

Resting BP was measured twice with one week interval; in each session measurement was repeated 3 times in a seated position using an aneroid sphygmomanometer (Erka, Germany) after 5 minutes of rest with 1-minute intervals. All measurements were performed in accordance with the criteria defined in the Eighth Report of the Joint National Committee on prevention, detection, evaluation, and treatment of high blood pressure (JNC 8).^8^ Patients with systolic/diastolic BP values >140/90 mmHg and/or receiving antihypertensive medication prescribed primarily for hypertension were considered to be hypertensive.

### SMMSE

SMMSE, is a standardized approach to score and interpret cognitive function among the elderly that provides a global score of cognitive ability in correlation with daily function.^9^,^10^ The reliability and validity of the Turkish version has been confirmed.^11^ SMMSE provides data on a multitude of cognitive domains, including orientation with regard to time and place (10 points), registration (3 points), attention and calculation (5 points), recalling (3 points), constructional ability, language, and the ability to understand and follow commands (9 points).^10^ A total score of 30 indicates no impairment and that of ≥26 is considered normal in the general population. Scores ranging from 21 to 25 indicate mild cognitive impairment. Patients who score between 20 and 10 have moderate cognitive impairment, whereas those who score between 9 and 0 have severe cognitive impairment.^10^ Cognitive data of all patients were collected by a single experienced researcher who had completed clinical training regarding SMMSE application in a neurology department in collaboration with expert neurologists.

### Statistical analysis

Statistical analysis was performed by the IBM Statistical Package for the Social Sciences Statistics for Windows, version 21.0 (SPSS, Armonk, NY, USA). Values were expressed as mean±SD, inter-quartile range (IQR), minimum–maximum, percent (%) or 95% confidence intervals (CI). Comparisons between subgroups were made using the Mann–Whitney U test owing to the non-normal distribution pattern of variables. Predicting effects of independent variables on scale scores were evaluated by mutually adjusted multivariate linear regression models. The limit of statistical significance was set at *p*-value less than 0.05.

## Results

### Demographics and clinical characteristics

Overall, 46.9% of the patients were aged ≥60 years old and 63.1% were females. All patients were primary school graduates. Obesity (BMI ≥30 kg/m^2^) was observed in 43.1% and hypertension in 76.9% of patients (**Table 1**).

**Table 1:** Demographics and clinical characteristics of Internal Medicine Outpatients.

Clinical characteristics	Mean±SD, n (%)
*Age (years)*	61.7±8.7
≤60	69 (53.1)
>60 and ≤70	39 (30.0)
>70	22 (16.9)
*Gender, n (%)*	
Female	82 (63.1)
Male	48 (36.9)
*Body mass index (kg/m^2^)*	28.9±4.9
<25	26 (20.0)
≥25 and <30	48 (36.9)
≥30	56 (43.1)
*Hypertensive patients*	100±76.9
SBP<140 mmHg and DBP<90 mmHg	46 (46.0)
SBP≥140 mmHg or DBP ≥ 90 mmHg	54 (54.0)
Age at onset of hypertension (years)	55.3±10.0
Duration of hypertension (years)	7.0±6.0
*Anti-hypertensive treatment*	
No treatment	15 (15.0)
Monotherapy	24 (24.0)
Combination therapy	61 (61.0)

DBP - diastolic blood pressure, SBP - systolic blood pressure, SD - standard deviation

### Standardized Mini-Mental State Examination scores and cognitive ability

The overall total SMMSE score was 25.3±3.4. Overall, 10% of patients showed moderate cognitive impairment, while 29.2% showed mild cognitive impairment (**Table 2**).

**Table 2:** Standardized Mini-Mental State Examination (SMMSE) scores and cognitive ability among Internal Medicine Outpatients.

SMMSE scores	Mean±SD
Total score	25.3±3.4
*Component scores*	
Orientation	4.1±1.0
Registration	3.0±0.2
Attention and calculation	4.1±1.3
Recalling	1.5±1.0
Language	7.5±0.7
Constructional ability	0.6±0.5
*Cognitive ability*	*n* (%)
Moderate cognitive impairment (scores 13-20)	13 (10.0)
Mild cognitive impairment (scores of 21-25)	38 (29.2)
Normal (scores ≥ 26)	79 (60.8)

### Standardized Mini-Mental State Examination scores with respect to study variables

The median (IQR) total SMMSE score was significantly higher in patients aged ≤60 years than in those aged >60 years old [27.0 (2.0) versus (vs) 25.0 (5.0), *p*<0.001], and the score did not differ significantly with respect to gender, the presence of obesity or hypertension, or anti-hypertensive treatment (**Table 3**). Besides attention and calculation, all component scores were lower (*p*=0.009 for orientation, *p*=0.008 for registration, and *p*<0.001 for recalling, language, and constructional ability) among older patients (**Table 3**).

**Table 3:** Standardized mini-mental state examination scores with respect to study variables among Internal Medicine Outpatients.

Study variables	Total	Orientation	Registration	Attention and calculation	Recalling	Language	Constructional ability
	Median (IQR)						
*Gender*							
Male, n=48	26.0 (4.0)	5.0 (1.0)	3.0 (0.0)	4.0 (1.0)	1.0 (1.0)	7.0 (1.0)	1.0 (1.0)
Female n=82	26.0 (4.0)	4.0 (2.0)	3.0 (0.0)	5.0 (1.0)	2.0 (1.0)	8.0 (1.0)	1.0 (1.0)
	*p*=0.690	*p*=0.002	*p*=0.498	*p*=0.434	*p*=0.044	*p*=0.183	*p*=0.455
*Age*							
≤60 years n=69	27.0 (2.0)	5.0 (1.0)	3.0 (0.0)	5.0 (1.0)	2.0 (1.0)	8.0 (1.0)	1.0 (1.0)
>60 years n=61	25.0 (5.0)	4.0 (2.0)	3.0 (0.0)	5.0 (1.0)	1. 0 (2.0)	7.0 (1.0)	0.0 (1.0)
	*p*<0.001	*p*=0.009	*p*=0.008	*p*=0.806	*p*<0.001	*p*<0.001	*p*<0.001
*BMI category*							
<25 kg/m^2^n=26	26.0 (6.0)	4.0 (1.0)	3.0 (0.0)	4.0 (1.0)	1.0 (1.0)	8.0 (1.0)	1.0 (1.0)
≥25 kg/m^2^n=104	26.0 (3.5)	4.0 (1.0)	3.0 (0.0)	5.0 (1.0)	2.0 (1.0)	8.0 (1.0)	1.0 (1.0)
	*p*=0.758	*p*=0.726	*p*=0.835	*p*=0.332	*p*=0.205	*p*=0.672	*p*=0.860
*Hypertension*							
Yes n=100	26.0 (4.0)	5.0 (1.0)	3.0 (0.0)	5.0 (1.0)	2.0 (1.0)	8.0 (1.0)	1.0 (1.0)
No n=30	27.0 (3.0)	4.0 (1.5)	3.0 (0.0)	4.0 (1.0)	2.0 (1.0)	8.0 (1.0)	1.0 (1.0)
	*p*=0.106	*p*=0.166	*p*=0.171	*p*=0.435	*p*=0.169	*p*=0.778	*p*=0.083
*Treatment*							
None n=15	26.0 (5.0)	4.0 (2.0)	3.0 (0.0)	5.0 (1.0)	1.0 (2.0)	7.0 (1.0)	0.0 (1.0)
Monotherapy n=24	26.0 (5.0)	4.0 (2.0)	3.0 (0.0)	5.0 (2.0)	2.0 (1.0)	7.0 (1.0)	1.0 (1.0)
Combination therapy n=61	27.0 (4.0)	4.0 (1.0)	3.0 (0.0)	5.0 (1.0)	2.0 (1.0)	8.0 (1.0)	1.0 (1.0)
	*p*=0.160	*p*=0.593	*p*=0.360	*p*=0.939	*p*=0.532	*p*=0.042	*p*=0.540

BMI - body mass index, IQR - interquartile range

Significantly higher median (IQR) scores in males than in females were noted for orientation [5.0 (1.0) vs. 4.0 (2.0), *p*=0.002], and language scores were higher in case of combination treatment than monotherapy or no therapy [8.0 (1.0) vs. 7.0 (1.0), *p*=0.042] among hypertensive patients. No significant difference was noted in the SMMSE component scores with respect to obesity and hypertension (**Table 3**).

### Multivariate linear regression analyses

Mutually adjusted multivariate linear regression analyses revealed that the female gender significantly decreased the total SMMSE score (B, −1.27; 95% CI, −2.36 to −0.18; *p*=0.023) and aging significantly decreased the total SMMSE score by 0.20 (B, −0.20; 95% CI, −0.26 to −0.14; *p*<0.001), whereas neither BMI nor being hypertensive turned out to be a significant predictor of the total SMMSE score (**Table 4**).

**Table 4:** Mutually adjusted multivariate linear regression analysis of factors potentially predicting SMMSE scores among internal medicine outpatients.

SMMSE score	Age (years)	*Gender	BMI	**Hypertension
	LR coefficient (%95 CI)			
Total	−0.20 (−0.26/−0.14)	−1.27 (−2.36/−0.18)	0.05 (−0.06/0.16)	−0.33 (−1.56/0.91)
*p-*value	<0.001	0.023	0.406	0.598
Orientation	−0.05 (−0.06/−0.03)	−0.72 (−1.04/−0.40)	0.01 (−0.02/0.05)	−0.18 (-0.55/0.19)
*p-*value	<0.001	<0.001	0.415	0.332
Registration	−0.01 (−0.01/−0.002)	0.01 (−0.06/0.09)	−0.004 (−0.01/0.004)	−0.03 (−0.12/0.05)
*p-*value	0.003	0.724	0.354	0.462
Attention and calculation	−0.01 (−0.04/0.01)	−0.07 (−0.55/0.41)	0.03 (−0.02/0.08)	0.04 (−0.51/0.58)
*p-*value	0.341	0.771	0.228	0.895
Recalling	−0.03 (−0.05/−0.02)	0.17 (−0.16/0.51)	0.02 (−0.02/0.05)	−0.22 (−0.61/0.16)
*p-*value	<0.001	0.308	0.293	0.248
Language	−0.02 (−0.04/−0.01)	0.07 (−0.17/0.31)	−0.01 (−0.03/0.02)	0.01 (−0.26/0.28)
*p-*value	<0.001	0.587	0.591	0.925
Constructional ability	−0.02 (−0.03/−0.01)	−0.12 (−0.29/0.05)	−0.01 (−0.03/0.01)	−0.09 (−0.29/0.10)
*p-*value	<0.001	0.176	0.300	0.351

BMI, body mass index; LR, linear regression; SMMSE, standardized Mini-Mental State Examination,

* Results for being female relative to being male,

** results for having hypertension relative to being normotensive

Aging (B, −0.05; 95% CI, −0.06 to −0.03; *p*< 0.001) and female gender (B, −0.72; 95% CI, −1.04 to −0.40; *p*< 0.001) significantly decreased the orientation score (**Table 4**). Aging was observed to significantly decrease registration score by 0.01 (B, −0.01; 95% CI, −0.01 to 0.00; *p*=0.003), recall scores by 0.03 (B, −0.03; 95% CI, −0.05 to −0.02; *p*<0.001), language scores by 0.02 (B, −0.02; 95% CI, −0.04 to −0.01; *p*<0.001), and constructional ability scores by 0.02 (B, −0.02; 95% CI, −0.03 to −0.01; *p*<0.001) (**Table 4**).

## Discussion

Our cohort cross sectional study of Internal Medicine Outpatients revealed mild-to-moderate cognitive impairment in more than one third of the patients. The Age was the most predominant predictor of the total and all-component SMMSE scores. Female gender was a significant determinant of low orientation and total scores, whereas hypertension and obesity had no significant impact on cognitive ability based on the total and all-component SMMSE scores. Old age was the most predominant predictor of cognitive ability and mild-to-moderate cognitive impairment was evident in more than one third of the patients. Although this appears consistent with the fact that almost half of the patients in our cohort are aged ≥60 years, it should also be noted that all the patients were primary school graduates. Given its relation to the acquisition and constant implementation of higher-order cognitive skills, low levels of education have been considered a risk factor for an earlier onset and higher prevalence of dementia.^12^ Low levels of cognitive reserve have been associated with an increased likelihood of clinical dementia,^13^ whereas higher education has been associated with high levels of cognitive reserve.^14^

A strong influence of schooling on the SMMSE scores was reported, particularly in mild-to-moderate rather than severe stages of dementia, possibly in association with the dependence of cognitive performance on factors such as previous formal education rather than functional aspects.^15^ Hence, it appears worth stressing the likely contribution of low educational status to the prevalence of cognitive impairment observed in our cohort study. Moreover, given the fact that patients with dementia were not included in the present cohort, our findings are in agreement with the available data on the utility of SMMSE in assessing cognitive impairment in patients with lower educational status, particularly in the case of mild degree cognitive dysfunction.^15^ Indeed evidence of better cognitive status in more than half the patients with low educational level in our cohort seems notable, given the increased likelihood of lower educated patients with better cognitive status to benefit from chronic disease self-management programs targeting improved health behavior and health-related quality of life.^16^ Despite the well-documented evidence on the association of hypertension with development of cognitive decline and dementia, and the role of high blood pressure in middle-aged population in predicting future risk of poor cognitive outcomes,^5^,^17^-^20^ our results revealed no significant impact of hypertension on cognitive ability in internal medicine outpatients. Nonetheless, given the inclusion of patients with new-onset hypertension in our cohort study, the presence of uncontrolled hypertension in half hypertensive patients, and the age-dependent decline shown in cognitive ability, the likelihood of accelerated cognitive impairment among hypertensive patients in the upcoming years cannot be ruled out based entirely on our findings.

Given the higher language scores noted in our hypertensive patients receiving combined antihypertensive therapy as compared with patients receiving monotherapy or no therapy, our findings may emphasize the role of appropriate antihypertensive treatment in improving the cognitive function among hypertensive patients.^18^,^19^ Furthermore, controlling BP levels has been suggested to protect cognitive functions in elderly individuals, particularly in females.^21^

In addition to association of female gender with low total and orientation domain scores of SMMS, the lack of ongoing anti-hypertensive treatment was associated with low scores in language domain of SMMSE, in our cohort. Hence, in addition to the protective effect of young age and male gender on overall cognitive functions among internal medicine outpatients with low educational status, our results emphasize the potential role of antihypertensive therapy in ameliorating domain-specific cognitive impairment among hypertensive patients.

Nevertheless, it should be noted that limited and conflicting data are available on the effect of antihypertensive therapy on cognitive function indicating their complex interaction with lack of clear evidence on the modifying role of antihypertensive treatment on the development or progression of cognitive impairment.^17^-^19^,^22^,^23^ Certain limitations to this study should be considered. First, the evaluation of cognitive function on the basis of SMMSE per se appears to be the major limitation of the present study. While SMMSE is the most widely used and easy to administer screening measure of cognition, it is insensitive to differentiate normal cognition from mild cognitive impairment in clinical practice.^24^ Nonetheless, it should be noted that it provides a global cognitive ability score that strongly correlates with daily living activities.^10^ Second, taking into account the likely heterogeneity in the levels of schooling, our findings may not be generalizable to the whole population of Turkish internal medicine outpatients.

In conclusion, our findings indicate the utility of SMMSE as a screening test among middle-aged and elderly internal medicine outpatients with low levels of formal education. The older age and female gender seems to be the predictors of an overall poor cognitive ability with the potential role of antihypertensive therapy in hypertensive patients in terms of ameliorating domain-specific cognitive impairment. There is a need for future larger scale studies in middle aged and elderly clinical populations addressing the influence of schooling on SMMSE scores with respect to severity of cognitive impairment along with the potential contribution of the duration and treatment of co-morbid hypertension.
